# A new locus on chromosome 22q13.31 linked to recessive genetic epilepsy with febrile seizures plus (GEFS+) in a Tunisian consanguineous family

**DOI:** 10.1186/1471-2156-14-93

**Published:** 2013-09-25

**Authors:** Nejla Belhedi, Frédérique Bena, Amel Mrabet, Michel Guipponi, Chiraz Bouchlaka Souissi, Hela Khiari Mrabet, Amel Benammar Elgaaied, Alain Malafosse, Annick Salzmann

**Affiliations:** 1 Laboratory of Genetics, Immunology and Human Pathologies, University Tunis el ManarTunisia, Tunis, 2092, Tunisia; 2 Neurological Department, Charles Nicolle Hospital, Tunis, Tunisia; 3 Department of Genetic Medicine and Laboratory, University Hospitals of Geneva, Geneva, Switzerland; 4 Department of Psychiatry, University of Geneva, Geneva, Switzerland

**Keywords:** Febrile seizures, GEFS+, Autosomal recessive, Genome wide SNPs, Linkage analysis, Exome sequencing

## Abstract

**Background:**

Genetic epilepsy with febrile seizures plus (GEFS+) is a familial epilepsy syndrome with extremely variable expressivity. The aim of our study was to identify the responsible locus for GEFS+ syndrome in a consanguineous Tunisian family showing three affected members, by carrying out a genome-wide single nucleotide polymorphisms (SNPs) genotyping followed by a whole-exome sequencing. We hypothesized an autosomal recessive (AR) mode of inheritance.

**Results:**

Parametric linkage analysis and haplotype reconstruction identified a new unique identical by descent (IBD) interval of 527 kb, flanking by two microsatellite markers, 18GTchr22 and 15ACchr22b, on human chromosome 22q13.31 with a maximum multipoint LOD score of 2.51. Our analysis was refined by the use of a set of microsatellite markers. We showed that one of them was homozygous for the same allele in all affected individuals and heterozygous in healthy members of this family. This microsatellite marker, we called 17ACchr22, is located in an intronic region of *TBC1D22A* gene, which encodes a GTPase activator activity. Whole-exome sequencing did not reveal any mutation on chromosome 22q13.31 at the genome wide level.

**Conclusions:**

Our findings suggest that *TBC1D22A* is a new locus for GEFS+.

## Background

Epilepsy is one of the most common serious neurological disorders at worldwide level [1]. Environmental and genetic factors are known to play a role in its pathogenesis. Segregation studies suggest that most epileptic syndromes are complex disorders, but several monogenic forms have also been described. Genetic (formerly named generalized [2]) epilepsy with febrile seizures plus (GEFS+) is such a Mendelian inherited epileptic syndrome. This familial autosomal dominant (AD) epilepsy shows a wide range of phenotypes such as febrile seizures (FS), FS plus (FS+) – defined as FS persisting beyond the age of 6 – as well generalized and partial seizures [2,3]. In some families, most severe epileptic phenotypes have been described such as the severe myoclonic epilepsy of infancy or Dravet syndrome [4-6].

Up to now, eight GEFS+ loci have been registered on OMIM database [7-16]. Three genes are known to be causative for GEFS+ phenotype: *SCN1B* (MIM#600235;GEFS+1) [7], *SCN1A* (MIM#182389; GEFS+2) [10] and *GABRG2* (MIM#137164; GEFS+3) [11]. Familial forms of pure febrile seizures (FEB) have also been described. Segregation and twin studies of FS suggest a polygenic or multifactorial mode of inheritance [17,18]. In most FEB families, phenotype follows an AD mode of inheritance [19]. To date, according to OMIM (Online Mendelian Inheritance in Man – http://www.ncbi.nlm.nih.gov/omim) database, eleven FEB loci have already been reported [20-30] and only four genes have been linked to FS related phenotypes: *SCN1A* (MIM#182389; FEB3A) [31], *MASS1* or *GPR98* (MIM# 602851; FEB4) [32], *GABRG2* (MIM#137164; FEB8) [27] and *CPA6* (MIM#609562; FEB11) [30]. Finally, two other genes are considered as “susceptibility” genes to FEB syndrome and epilepsy: *GABRD* (MIM#137163; GEFS+5) [15] and *SCN9A* (MIM#603415; FEB3B/GEFS+7) [33].

In the present study, we reported the clinical and genetic studies of a GEFS+ consanguineous Tunisian family with an autosomal recessive (AR) mode of inheritance. We used homozygosity mapping, which is a method of choice for localizing genes responsible for AR diseases in consanguineous families [34]. This approach allows identifying identical by descent (IBD) regions inherited from a common ancestor. IBD loci are homozygous in all the affected members but not in other relatives. In the present study we report a new locus on chromosome 22q13.31 in which *TBC1D22A* gene (TBC1 domain family, member 22A, also known as *C22orf4*) is located.

## Results

### Clinical description of consanguineous Tunisian family with GEFS+ patients

**Figure 1:**
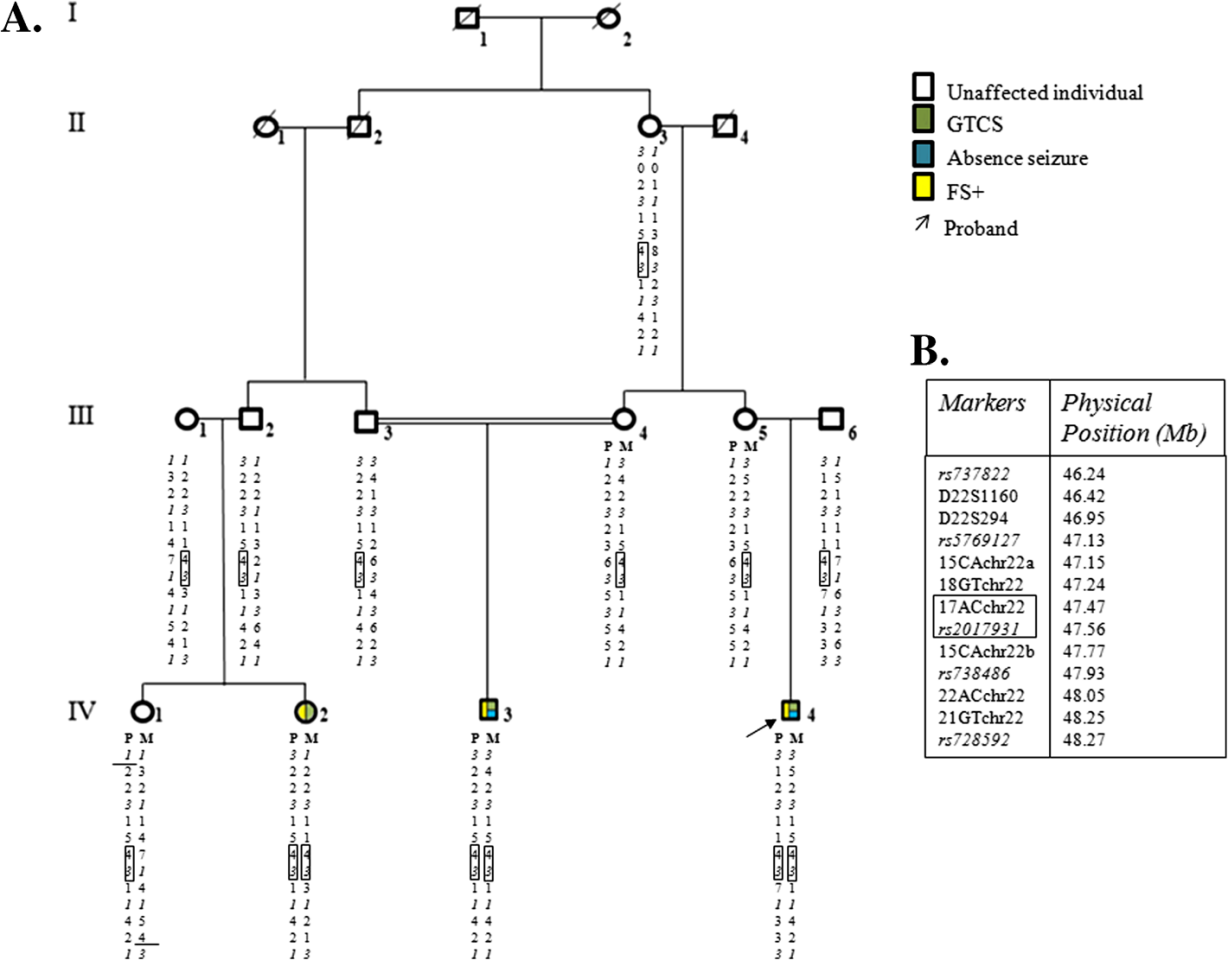
**Consanguineous Tunisian GEFS+family pedigree.** Legend: **A.** Haplotype reconstruction for markers on chromosome 22q13.31, which are ordered from centromere to telomere. Markers from the Illumina array are in italic. The IBD haplotype shared by family members is shown in solid lines. Recombinaison events are shown by solid drawbar. Maternal and paternal chromosomes are designated by M and P, respectively. Each phenotype is describing by different colors. **B.** List of markers used for haplotype reconstruction with physical position. Markers from the Illumina array are in italic.

The familial pedigree is shown in Figure 1 and main clinical features of affected individuals are described in Table 1. The occurrence of generalized tonico clonic seizures (GTCS), absence seizure and FS+ led us to consider a GEFS+ syndrome in the present family.

**Table 1:** Clinical characteristics of affected individuals

Subject (sex, age) ^*^	**FS**		**AFS**		**EEG**	**Neurological examination**	**MRI**	**Treatment/age**
	**Onset age/remission, age**	**n**	**Onset age/remission, age**	**Type/n**				
IV-2 (F, 13 y)	4 y/yes, 10 y	4	7 y/8 y	GTCS/3	Normal	Normal	Normal	VPA/7 y-until now
IV-3 (M, 18 y)	2 y/yes, 13 y	Numerous	3 y/7 y	GTCS/1 yearly	Normal	Normal	Normal	VPA/8 y-12 y
			8 y/9 y	A/numerous				
IV-4 (M, 17 y)	2 y/no	Numerous	6 y/11 y	GTCS/5	3 Hz generalized SW	Mild mental retardation	Normal	VPA/5 y-until now
				A/numerous				

^*^All individuals were agree to publish clinical details.

*M*: Male, *F*: Female, *y*: years, *FS*: Febrile Seizures, *AFS*: Afebrile Seizures, *n*: Seizures number, *EEG*: Electroencephalogram, *GTC*: Generalized Tonico Clonic Seizures, *A*: Absence, *SW*: Spike Wave, *MRI*: Magnetic Resonance Imaging, *VPA*: Valproic Acid.

The proband (IV-4) is a 17-year-old boy, born at term after an uneventful pregnancy. At the age of 2, he suffered from a first complex FS. At the age of 6, a febrile GTCS and absence seizures were observed. Due to the continuation of FS, since the age of 5, he has been treated with valproic acid. Neurological examination revealed a mild mental retardation. Standard EEG tracings showed generalized spike waves.

His first cousin, patient IV-3, is an 18-year-old boy, born at term after an uneventful pregnancy, from a consanguineous marriage between first degree cousins. In addition to FS+ which persisted until the age of 13, he experienced, at the age of 3, GTCS. Since the age of 8, absence seizures were also observed. Between the ages of 8 to 12, he was treated with valproic acid. Neurological and EEG exams were normal.

Patient IV-2, first cousin of the two previously described patients, is a 13-year-old girl, born at term after an uneventful pregnancy. She showed FS+ until the age of 10 and she suffered from GTCS since the age of 7. She has been treated by valproic acid since the age of 5. Neurological examination and EEG tracings were normal.

MRI scans were normal in all affected family members.

### Genetic linkage and haplotype analysis

We assessed microsatellite markers (Additional file 1: Table S1) of four GEFS+ loci and seven FEB loci, that were known at that time. Haplotype reconstructions and analysis of recombination events allowed excluding all the GEFS+ and FEB loci analyzed in the present family.

A genome wide genotyping was then performed and parametric linkage using the AR mode of inheritance identified a linked region of 10.6 Mb on chromosome 22q13.31 (Additional file 2: Figure FS1) flanked by the two SNPs rs3203726 and rs728592 with a maximum multipoint LOD score of 2.51 (Additional file 1: Table S5). On the same chromosome, positive LOD scores were also observed at six other loci (Additional file 1: Table S6). However, haplotype analysis showed that all these loci exhibited only one or two consecutive homozygous SNPs in every affected individual. Consequently, they are less likely to represent the disease locus. Moreover, due to the number of obligate carriers of the disease (8) compared to affected ones (3), the AD model is not likely.

**Figure 2:**
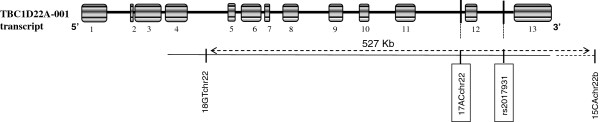
**Structure of the *TBC1D22A *gene according to Ensembl (**http://www.ensembl.org/index.html**) with localization of markers within and flanking the 527 Kb IBD linked locus.**

The 10.6 Mb candidate region was further confirmed by using a set of microsatellite markers (Additional file 1: Table S2). For haplotype reconstructions, we used homozygosity mapping method, which allow localizing homozygous IBD genomic region(s) responsible for AR diseases in consanguineous families. We confirmed the existence of a common homozygous IBD haplotype shared by all the three patients. Therefore, the candidate region was reduced to an interval of 527 kb, flanked by two microsatellite markers, 18GTchr22 and 15ACchr22b. This locus was homozygous in all three affected relatives and heterozygous in other family members (Figure 1A). Within this homozygous IBD locus lie the 9 last exons and the 3’UTR of *TBC1D22A* which encodes the member 22A of Rab GTPase activator with a TBC1 domain family (Figure 2). Two polymorphic markers, 17ACchr22 and rs2017931, are also displayed within the IBD interval (Figure 1A). The most interesting one is the 17ACchr22 microsatellite marker due to its high variability. Its allele 4, corresponding to 16 AC repeats, was found homozygous in all affected relatives and heterozygous in all other unaffected family members. We estimated allele frequencies of 17ACchr22 in a sample of 70 Tunisian controls. Ten alleles (corresponding to 13 to 22 AC repeats) were observed, and the most common alleles were allele 7 (19 repeats) 25%, allele 8 (20 repeats) 22.1%, allele 5 (17 repeats) 17.9%, and allele 6 (18 repeats) 10.7%. Finally, the IBD allele 4, had a frequency of 7.14% and was never found homozygous in the present Tunisian population (Additional file 1: Table S7).

Array CGH analysis did not reveal any CNV at the genome-wide level in affected individual IV-3.

### Mutational analysis

Mutation analysis was performed by whole-exome sequencing in patient IV-3 where 94.5% of the coding part of the RefSeq genes was covered at least 8×, which was sufficient to detect homozygous substitutions. This experiment revealed only three homozygous non-synonymous putative deleterious variations over the entire genome. Two of them were found on chromosome 22q13.31, within the 10.6 Mb linked region: c.5159C > T (S1720L) in the *TNRC6B* (trinucleotide repeat containing 6B) gene and c.4433C > T (T1478M) (rs8141262) in the *CACNA1I* (alpha 1I subunit of calcium voltage-dependant channel) gene. Genotyping these two missense variations, in all Tunisian family members, was done by HRM assay and confirmed by Sanger sequencing. These analyses showed that they are not linked to the disease phenotype, since the two other affected cousins (IV-2 and IV-4) were heterozygous for both variations. The third variation, found homozygous in patient IV-3, is located on chromosome 1p36.11: c.604T > C (S202P) (rs6687605) in the *LDLRAP1* (low density lipoprotein receptor adaptor protein 1) gene. This polymorphism, genotyped by Sanger sequencing (Additional file 1: Table S3), was also found homozygous in his unaffected cousin IV-1. Therefore, rs6687605 is not linked to GEFS+ phenotype in this family, according to the IBD assumption. Finally, we also performed Sanger sequencing for *KCNJ4*, since this gene codes for a ion channel protein. This family of genes are often found mutated in several epilepsies. Moreover, this gene is located within one of the 6 chromosome 22 short homozygous regions (Additional file 1: Table S6), which are considered as not linked to the disease (see Genetic linkage and haplotype analysis section). No mutation was observed in this gene.

## Discussion

The present study described clinical and molecular investigations on GEFS+ syndrome in a Tunisian consanguineous family. The occurrence of both GTCS and absence in addition to FS+, and in absence of other types of seizures (e.g. myoclonic jerks), led us to consider that GEFS+ phenotype is likely within this family [2]. To our knowledge, it would be the first description of a consanguineous GEFS+ Tunisian family with a putative AR mode of inheritance, a transmission previously described in a Moroccan consanguineous family, showing FS and temporal lobe epilepsy phenotypes [30]. Moreover, all GEFS+ families, described in OMIM database, exclusively show an AD mode of inheritance [7-16]. Using homozygous linkage and whole-exome sequencing method, we mapped the pathogenic locus responsible for the disease within a short interval of chromosome 22q13.31 in the present Tunisian consanguineous family.

We first excluded by linkage analysis all known GEFS+ loci at that time, as well as those associated to FS phenotypes. Epileptic syndromes have also been observed in patients with structural variants [35,36], such as CNVs on chromosome 15q13.3 are known to play an important role in the genetic etiology of idiopathic generalized epilepsy [37]. We also excluded such genomic event in this family.

The 22q13.31 region, where we mapped the disease locus, has been implicated in a complex phenotype with epilepsy in 5-year-old boy with a 7.9 Mb *de novo* deletion of chromosome 22q13.2-qter [38].

Based on the homozygosity of an IBD locus in patients only, refined mapping allowed reducing the disease locus to the 9 last exons and 3’UTR of *TBC1D22A*, and even to a shorter interval around the exon 12. However no mutation has been observed by whole-exome sequencing in any of these last exons of *TBC1D22A*. Homozygous alleles of the two polymorphic markers (17ACchr22 microsatellite and rs2017931), found in all patients, are likely to be non-functional and probably in linkage disequilibrium with the disease mutation in one of the *TBC1D22A* introns or in 3’UTR. The disease mutation could affect the splicing of *TBC1D22A*. Unfortunately, we were unable to test this hypothesis in absence of available RNA samples from the affected members of the family.

The first crystal structure of TBC1D22A was done in 2008 showing the diversity of human TBC domain family members [39]. Very recently, Shapshak et al. found that *TBC1D22A* gene had a differing gene expression profile across patients showing HIV associated dementia [40]. *TBC1D22A* gene is expressed in brain tissues with a percentage of 2.14% (http://smd.princeton.edu/cgi-bin/source/sourceSearch). Moreover, GWAS suggested an association with human longevity and a SNP (rs5766691) located in *TBC1D22A*[41], within the 527 kb IBD homozygous interval.

## Conclusions

In conclusion, we reported a new locus on chromosome 22q13.31 in a consanguineous Tunisian family with a GEFS+ phenotype with an original AR mode of inheritance. We were not able to identify the disease mutation but restricted the linked interval to a part of *TBC1D22A*, which is expanded from exon 5 to the 3’UTR of the gene. Further studies will confirm whether this gene is also mutated in other families with GEFS+.

This study was approved by the Research and Ethics Review Board of the Department of Neurology, University Hospital of Geneva and by the Ministry of Higher Education and Scientific and Technological Research of Tunisia.

## Methods

### Family ascertainment and phenotyping

We recruited a consanguineous Tunisian family extended over four generations, comprising 3 affected and 19 non-affected members, who all originated from Tunis (Tunisia) (Figure 1). The segregation of the trait suggests an AR mode of inheritance: all the affected relatives are on the last generation and one of them (IV-3) is born from first cousins (III-3 and III-4). The two other affected relatives were born from one parent with the same common ancestors as III-3 and III-4. The two other parents (III-1 and III-6) could descend from a putative common ancestor with their spouses because they all originated from a small village located in the North of Tunisia.

All family members were agree to publish clinical details and they were clinically assessed at Charles Nicolle University Hospital (Tunis) by experienced epileptologists (Hela Khiari-Mrabet and Amel Mrabet). Information on FS and afebrile seizures, age at onset, duration, type and number of seizures, intellectual outcome, antiepileptic drug therapy, and seizures outcome were obtained from their parents and case notes when available. Electroencephalographic (EEG) and magnetic resonance imaging (MRI) scans were done for all affected family members. FS+ phenotype was defined as FS persisting beyond the age of 6 years [2]. Generalized tonic clonic seizures (GTCS) and absence seizures were defined according to the criteria established by the “International League against Epilepsy” [42-44].

### Tunisian control group

Seventy unrelated healthy Tunisian controls (mean age 48.5 ± 16.5) were recruited at Charles Nicolle University Hospital (Tunis). These individuals did not have any personal and familial history of seizures.

### Genomic DNA

Oral and written informed consent was obtained from all participants or their legal representatives. Blood samples were collected and genomic DNA was extracted from peripheral blood leucocytes by phenol/chloroform procedure [45].

### Exclusion mapping

Linkage analysis with a panel of 36 microsatellite markers spanning the FEB loci (FEB1, FEB2, FEB3, FEB4, FEB5, FEB6 and FEB11/ETL5) and GEFS+ loci (GEFS+1, GEFS+2, GEFS+3 and GEFS+4) known at that time were firstly explored (Table 2). Primer sequences are listed on Additional file 1: Table S1 and PCR conditions are available as Additional file 1. We excluded all these previously linked loci by linkage analysis.

**Table 2:** Tested and excluded known FS and GEFS+ loci

**Locus**	**Candidate gene**	**Marker**	**Genetic position (cM)**	**Physical position(Mb)**	**LOD Scores**
FEB1		D8S553	81.50	67.10	-9.31
		D8S1058	90.10	73.09	-10.43
		D8S279	90.20	73.15	-15.94
FEB2		D19S424	10.97	3.18	-16.30
		D19S177	20.75	5.47	-12.04
		D19S1034	20.75	6.06	-8.99
		D19S406	25.17	7.33	-13.27
		D19S76	25.17	7.56	-9.23
FEB4	*MASS1*	MASS1Int85	99.30	90.20	-12.83
		D5S644	104.76	95.84	-12.95
FEB5		D6S1620	129.10	129.99	-5.31
		D6S472	132.70	132.58	-9.52
FEB6	*IMPA2*	D18S1153	34.70	10.12	-10.08
		IMPA2Int5	42.00	12.01	-16.96
		D18S71	42.80	12.59	-9.78
GEFS+1	*SCN1B*	D19S425	58.70	40.19	-11.14
		SCN1BInt1	59.00	40.21	-11.52
		D19S893	61.40	40.26	-11.43
GEFS+2/FEB3	*SCN1A/SCN2A*	D2S2330	175.50	166.41	-10.71
		D2S2345	177.20	168.43	-6.59
		D2S2314	188.90	176.57	-5.54
GEFS+3	*GABRG2*	D5S1465	162.00	161.35	-12.02
		GABRG2Int1	162.50	161.44	-17.29
		GABRG2Int5	162.60	161.48	-17.29
		D5S2576	162.63	161.51	-16.17
		D17S2131	162.75	161.88	-15.99
		D5S422	163.90	162.09	-14.19
GEFS+4		D2S1360	38.33	17.36	-3.68
		D2S305	38.87	19.28	-3.69
		D2S2342	40.47	20.19	-3.81
FEB11/ETL5	*CPA6*	D8S507	75.00	59.30	-5.18
		D8S1812	78.30	60.85	-5.47
		D8S1843	78.80	62.42	-9.12
		D8S544	81.00	65.75	-11.07
		D8S533	81.50	67.16	-11.37
		D8S1775	85.80	68.99	-11.99

### Genetic linkage and haplotype analysis

A genome-wide linkage study was performed by using the Illumina Gene Chip Linkage Infinium II HumanLinkage-24 Panel Breadchip® (Illumina, San Diego, CA), which allowed us to genotype 5913 SNPs.

Two-point and multipoint logarithm of odds (LOD) scores were calculated with MERLIN 1.1 program, assuming an AR inheritance with complete penetrance, a disease allele frequency of 0.00001 and a phenocopy rate of 0. The haplotype reconstruction for family members was done manually, regardless of the individual affection status.

To confirm the region with a LOD score of 2.51 found by linkage analysis and in order to refine the mapping, we chose a set of 8 microsatellite markers located at the chromosome 22q13.31 and distributed at average intervals of 0.2 Mb (Figure 1B). According to the linked region defined by positive LOD score, we chose some microsatellite repeats using UCSC database (http://genome.ucsc.edu) by specifying “microsatellite” in “variations and repeats” part. Primer sequences (Additional file 1: Table S2) and PCR conditions are available on Additional file 1.

### Genome wide CNVs analysis

We have also performed a copy number variations (CNVs) analysis by using the Agilent Human Genome comparative genomic hybridization (CGH) Microarray Kit 244 K (Agilent, Santa Clara, CA). The slide was scanned on an Agilent DNA microarray scanner. Data were obtained by Agilent Feature extraction software 9 and analyzed with Agilent CGH analytics 3.4 software, using the statistical algorithms zscores and ADM-2 with a sensitivity threshold of 2.5 and 6.0, respectively, and a moving average window of 0.2 Mb. Mapping data were analyzed on the human genome sequence using the NCBI database Build 35, Hg17 (http://www.ncbi.nlm.nih.gov).

### Candidate gene mutational analysis

Linkage results showed six other loci with positive LOD scores in the vicinity of the 527 kb IBD region. In one of them lies *KCNJ4*, which encodes a potassium inwardly-rectifying channel (subfamily J and member 4). According to its physical position and its functional role, this putative candidate gene for epilepsy, was sequenced in the present consanguineous family. We performed PCR for the two exons and splice junctions. We designed flanking exon primers from published sequences with the Primer3 (version.0.4.0) online program (http://bioinfo.ut.ee/primer3-0.4.0/primer3/input.htm). Primer sequences are listed on Additional file 1: Table S3 and PCR conditions are available on Additional file 1.

### Exome sequencing

Paired-end sequencing was performed using Illumina GAIIx/HiSeq 2000 instruments (Illumina, San Diego, CA) available at the Department of Genetic Medecine of the School of Medicine from Geneva. Sequence capture was performed using Agilent Sure Select Technology (Agilent, Santa Clara, CA) using either the All Exome kits or custom design corresponding to the linkage intervals and IBD regions. We typically obtain in excess of 30 million read pairs (60 million reads) of 76 nucleotides per GAIIx lane and 100 million reads pairs of 100 bp per HiSeq2000 lane allowing multiplexing of 2–3 exomes per lane.

Alignment of reads and call of single nucleotide variants and small indels were done using the latest version of MAQ/BWA [46] and PinDel [47] softwares. Only genetic variants with high-quality score were further investigated. Variant annotation was performed using the ANNOVAR package [48]. Generation of high-quality data necessary for reliable SNP detection across the targeted regions required 30-fold genomic coverage (equivalent to 15-fold coverage per haplotype).

Each DNA variant was subsequently compared to Single Nucleotide Polymorphism database (dbSNP132, NCBI) and 1000genome project dataset. Only the unique and novel DNA variants were selected for additional filtering based on the nature of the mutation (gene structure location, nucleotide conservation, codon change, de novo occurrence) and familial segregation. Polyphen2 [49] and Sift [50] programs were used to assess the pathogenecity of the filtered variants. If necessary, the final list of potential pathogenic variants were further refined by selecting genes functionally relevant to epilepsy. T1478M of *CACNA1I*, S1720L of *TNRC6B* and S202P of *LDLRAP1* were further explored by classic Sanger sequencing and high resolution melt (HRM) assay using a Rotor-Gene 6000 instrument (Corbett Life Science, Australia). More details are given in Additional file 1 and in Additional file 1: Table S3 and Additional file 1: Table S4.

## Competing interests

The authors declare that they have no competing interests.

## Author’s contributions

NB designed the study and wrote the manuscript. FB performed the genome wide CNVs analysis. MG performed the exome sequencing analysis. CBS interpreted the clinical data. HKM and AMr performed the clinical study. ABAE, AM and AS performed supervision of the present study and critical reading of manuscript. All authors have read and approved the final manuscript.

## Author’s information

Nejla Belhedi (NB): PhD student; Laboratory of Genetic Immunology and Human Pathologies University Tunis el ManarTunisia and Neurological Department Charles Nicolle Hospital Tunis Tunisia. Frederic Bena (FB): MD, FAMH; Department of Genetic Medecine and Laboratory University Hospitals of Geneva Switzerland. Michel Guipponi (MG): PhD; Department of Genetic Medecine and Laboratory University Hospitals of Geneva Switzerland. Chiraz Souissi-Bouchlaka (CSB): PhD; Laboratory of Genetic Immunology and Human Pathologies University Tunis el ManarTunisia. Hela Khiari-Mrabet (HKM): MD; Neurological Department Charles Nicolle Hospital Tunis Tunisia. Amel Mrabet (AMr): MD; Neurological Department Charles Nicolle Hospital Tunis Tunisia. Amel BenAmmar Elgaaied (ABAE): PhD; Laboratory of Genetic Immunology and Human Pathologies University Tunis el ManarTunisia. Alain Malafosse (AM): MD, PhD; Department of Genetic Medecine and Laboratory University Hospitals of Geneva and Department of Psychiatry University of Geneva Switzerland. Annick Salzmann (AS): PhD; Department of Psychiatry, University of Geneva, Switzerland.

## Supplementary Material

Additional file 1: Table S1 Primer Sequences of Microsatellite Markers for Exclusion Mapping. **Table S2** Primers Sequences of Microsatellite Markers for Haplotype Analysis on 22q13.31. **Table S3** Primers Sequences for Sanger Sequencing. **Table S4** Primers Sequences for HRM assay. **Table S5** Lod Scores of linked locus on chromosome 22q13.31. **Table S6** Loci on Chromosome 22q13.31 with Positive LOD Score. **Table S7** 17ACchr22 Microsatellite Genotype and Allele Distributions in the Tunisian Control Population. Click here for file

Additional file 2: Figure FS1 LOD scores were calculated using Merlin program. A new region was linked on the chromosome 22q with a maximum of LOD score of 2.51. Click here for file
