# Supplementary material for: A new locus on chromosome 22q13.31 linked to recessive genetic epilepsy with febrile seizures plus (GEFS+) in a Tunisian consanguineous family
Source: BMC Genet. 2013 Sep 25;14:93. doi: 10.1186/1471-2156-14-93 (PMC3851042; doi:10.1186/1471-2156-14-93)
Supplement: Additional file 1: Table S1 — Primer Sequences of Microsatellite Markers for Exclusion Mapping. Table S2 Primers Sequences of Microsatellite Markers for Haplotype Analysis on 22q13.31. Table S3 Primers Sequences for Sanger Sequencing. Table S4 Primers Sequences for HRM assay. Table S5 Lod Scores of linked locus on chromosome 22q13.31. Table S6 Loci on Chromosome 22q13.31 with Positive LOD Score. Table S7 17ACchr22 Microsatellite Genotype and Allele Distributions in the Tunisian Control Population. [file 1471-2156-14-93-S1.doc]

**Additional file 1**

PCR Conditions

*Exclusion Mapping*

PCR reactions were performed in a 25 µl volume containing: 100 ng of DNA, 0.5 µM of forward and reverse primers, 200 mM dNTPs (Invitrogen^TM^, Eugene, OR), 1X PCR buffer (Qiagen®, Germantown, MD) and 0.5 U of Taq Hot Start DNA polymerase (Qiagen®, Germantown, MD). DNA amplification reactions were performed in a thermocycler (Biometra, Germany) and were as followed: 95 °C for 3 min , 30 cycles of 94°C for 30 s , X°C for 30 s (X = annealing temperature ranged from 54°C to 60°C depending on the microsatellite marker amplified, see *Table S1*) and 72°C for 30 s. Primers sequences are described on *Table S1*. PCR products were run on a 10% polyacrylamide gel and marker alleles were visualized by ethidium bromide (BET).

*Haplotype analysis*

PCR reactions were performed in a 25 µl volume containing: 100 ng of DNA, 0.5 µM of forward and reverse primers, 200 mM dNTPs (Invitrogen^TM^, Eugene, OR), 1 x buffer (Qiagen®, Germantown, MD) and 0.5 U of Taq Hot Start DNA polymerase (Qiagen®, Germantown, MD). For 17ACchr22 and 22ACchr22 amplification’s reactions, we add 5 µl of Q solution (Qiagen®, Germantown, MD). DNA amplification reactions were performed in a thermocycler (Biometra, Germany) and were as followed: 94°C for 15 min; 1 cycle of 94 °C for 30 s, 62 °C for 30 s and 72 °C for 30 s, followed by 9 touch-down cycles, where the annealing temperature is reduced by 1 °C at each cycle, final step is 20 cycles of 94 °C for 30 s, 53 °C for 30 s and 72 °C for 30 s. Primers sequences are described on *Table S2*. PCR products were run on a 10% polyacrylamide gel and marker alleles were visualized by BET.

For 17ACchr22 microsatellite marker genotyping, ten alleles were found. Allele 1 ((AC)13) was 160 nucleotides (nt), allele 2 ((AC)14) was 162 nt, allele 3 ((AC)15) was 164 nt, allele 4 ((AC)16) was 166 nt, allele 5 ((AC)17) was 168 nt, allele 6 ((AC)18) was 170 nt, allele 7 ((AC)19) was 172 nt, allele 8 ((AC)20) was 174 nt, allele 9 ((AC)21) was 176 nt and allele 10 ((AC)22) was 178 nt .

*Mutational Analysis*

*KCNJ4* gene screening was performed using Sanger sequencing. PCR reactions were performed in a 50 µl volume containing: 200 ng of DNA, 1 µM of forward and reverse primers, 200 mM dNTPs (Invitrogen^TM^, Eugene, OR), 1 x buffer (Qiagen®, Germantown, MD) and 1 U of Taq Hot Start DNA polymerase (Qiagen®, Germantown, MD). DNA amplification reactions were performed in a thermocycler (Biometra, Germany) and were as followed: 94°C for 15 min; 1 cycle of 94 °C for 30 s, 62 °C for 30 s and 72 °C for 30 s, followed by 9 touch-down cycles, where the annealing temperature is reduced by 1 °C at each cycle, final step is 20 cycles of 94 °C for 30 s, 53 °C for 30 s and 72 °C for 30 s. Primers sequences are described in *Table S3*. PCR products were purified with QIAquick PCR Purification kit (Qiagen, Germantown, MD) and sequenced with an ABI PRISM 3100 Genetic Analyzer (PE Applied Biosystems, Foster City, CA)

Candidate variants T1478M of *CACNA1I* and S1720L of *TNRC6B* were firstly explored by high resolution melt (HRM) assay using a Rotor-Gene 6000 instrument (Corbett Life Science, Australia). Primers were designed according to the manufacturer’s instructions (*Table S4*). Amplicon sequence was analyzed by the Poland melting software program to predict melting behaviour (http://www.biophys.uni.-duesseldorf.de/local/POLAND//poland.html) and secondary structures were checked by DINAMelt (http://www.bioinfo.rpi.edu/ applications/hybrid/twostate-fold.php). PCR reaction was carried out with 100 ng of genomic DNA using Kappa 2G Robust Hot Start Kit (Kappa Biosystem, Cape Town, SA) in a 20 μl reaction mix containing 1 x buffer, 0.2 mM dNTPs, 7.5 mM of each primer, 0.01 mM Hot Start DNA polymerase and 0.04 μM EvaGreen fluorescent intercalating dye (Quantace, London, United Kingdom). Ampliﬁcation conditions were as follow: 95◦C for 10 min, 50 cycles of 95◦C for 5 sec, 60◦C for 30 sec, and 72◦C for 20 sec. Alleles were identiﬁed by HRM set from 68◦C to 90◦C, with the temperature rising by 0.2◦C per second.

To confirm HRM results a Sanger sequencing was done. For T1478M of *CACNA1I* amplification reaction was performed in a 50 µl volume containing: 200 ng of DNA, 1 µM of forward and reverse primers, 200 mM dNTPs (Invitrogen^TM^, Eugene, OR), 1 x PCR buffer (Qiagen®, Germantown, MD) and 1 U of Taq Hot Start DNA polymerase (Qiagen®, Germantown, MD) and 1.4 µl of DMSO. Primers sequences are described in *Table S5*. DNA amplification reactions were performed in a thermocycler (Biometra, Germany) and were as followed: 94°C for 15 min; 1 cycle of 94 °C for 30 s, 62 °C for 30 s and 72 °C for 30 s, followed by 9 touch-down cycles, where the annealing temperature is reduced by 1 °C at each cycle, final step is 20 cycles of 94 °C for 30 s, 53 °C for 30 s and 72 °C for 30 s. We have also sequenced the S202P variant located in *LDLRAP1* gene on chromosome 1p36.11. For S1720L of *TNRC6B* and S202P *LDLRAP1* amplification, PCR reactions were performed in a 50 µl volume containing: 200 ng of DNA, 1 µM of forward and reverse primers, 200 mM dNTPs (Invitrogen^TM^, Eugene, OR), 1 x PCR Buffer A (Kappa Biosystem, Cape Town, SA) and 1 U of Kappa 2G Robust Hot Start Taq DNA polymerase (Kappa Biosystem, Cape Town, SA). DNA amplification reactions were performed in a thermocycler (Biometra, Germany) and were as followed: 95 °C for 3 min, 30 cycles of 94°C for 30 s, 58°C for 30 s and 72°C for 30 s. PCR Primer sequences are described in *Table S5*.

All PCR products were purified with QIAquick PCR Purification kit (Qiagen, Germantown, MD) and sequenced with an ABI PRISM 3100 Genetic Analyzer (PE Applied Biosystems, Foster City, CA)

**Additional file 1: *Table S1* *Primer Sequences of Microsatellite Markers for Exclusion Mapping***

| Locus | Microsatellite | Primer Sequences | | T° Annealing (°C) |
| --- | --- | --- | --- | --- |
|  |  | Forward | Reverse |  |
| FEB1 | D8S553 | GACGGCAGAATGAAACCTTG | TTACAGATTGCTCCTCCCTG | 58 |
|  | D8S1058 | GAAAGCATGAATAGCACCGAAC | CTTTTTGTGCCGGATTTGTAAAC | 58 |
|  | D8S279 | GTCATTCCCTCTTCCTCTC | GGGTGGATTGCAGAGAATTTG | 58 |
| FEB2 | D19S424 | CTGGTTATCTTGAGGGAGG | GGAGGAGTTAGATTTTTCAGG | 60 |
|  | D19S177 | TTGCACCACTGCACTCCAG | CCAATGACTTCAAGCACTAAG | 60 |
|  | D19S1034 | AGCACCTAGCAAATAGTAGG | GTAGACAGAGCAAGAACCTG | 60 |
|  | D19S406 | CCTCACGGATACCAATGGG | TCAGCTTGTCAATGTGGGG | 58 |
|  | D19S76 | CTGTTCACAAGGTGACAAGG | GTGACAGAGCCAGACTCTG | 62 |
| FEB4 | MASS1Int85 | CCAGCTCTACTTTCTACTTC | CTCCAGATTCAAGCAGAATC | 54 |
|  | D5S644 | CTAACTGGTAGATCAATGTGCC | CTACCTAGACCAAGGAAGCC | 60 |
| FEB5 | D6S1620 | AAAATCCCTTTTAGGATGTACCC | TGCTTCATCCTAAGTTAATGCAC | 60 |
|  | D6S472 | GACCCAAAAGATGTAATGGG | AAGTGACATAAGAGTGAGGG | 54 |
| FEB6 | D18S1153 | CAATCCTTGCCCTATGCATC | CAGATGGCAGCAGTGATTTC | 58 |
|  | IMPA2Int5 | TGACAGAATGGGGCTCCATC | CCTATATTTACCATATCTTAGGTC | 54 |
|  | D18S71 | CTTCCCTCTAGTGTCTCTTTC | TCAGAATGGGGGATCACTTTC | 58 |
| FEB11/ETL5 | D8S507 | TTCCTCAGAGCAGTTCAAAG | TAATCTTGCCCCAGTGAGAG | 50 |
|  | D8S1812 | AGAAGGCACCACTAATCC | TTTGGAAGGCAAAACA | 53 |
|  | D8S1843 | GCAACACATGTTGTCAAACCA | TGGTGCCTGCTCTTTTCTTT | 55 |
|  | D8S544 | GCCATTATGCTGTCTTGC | CTCTTGTGGAATTTGAGGG | 50 |
|  | D8S533 | CTTTGCCAGGGTGTTCAGAG | AGAGCCTTGTTTCATGGGAC | 55 |
|  | D8S1775 | TGGCAAATACACACTCTGCT | CCAAATAGGCTGATGAGAAACT | 55 |
| GEFS+1 | D19S425 | CAGAGCAACAGCATCATAGG | TTGCCATGTGACTGTAGCAG | 58 |
|  | SCN1BInt1 | TTCCCCCCGCGGTGATTCATC | ACATCCGCTAGGTGCACCGACC | 58 |
|  | D19S893 | ATTAATCCTGAGACTGGGGG | CTAACCCTTGTCTTTGGGG | 56 |
| GEFS+2/FEB3 | D2S2330 | AGCAAGCCCTTACAATAAGTCCC | CCTTTGTAGCTTGTCTCTGGCTG | 58 |
|  | D2S2345 | GTTCTTTTCTATGAAGACCTG | GCTCAGTAGAAATAGTGATTCTG | 58 |
|  | D2S2314 | GGTGTCAGTGAGACCCTGT | ATTTCTAGCGGCCCTAAAAC | 60 |
| GEFS+3 | D5S1465 | GAAATGACTAAATTTGGACCCTGCAG | CTGCGATTTGTAAAGTATAGATTGCAG | 54 |
|  | GABRG2Int1 | CAGTTAGATGAAGTGTTTTCC | GAAGCACAATAAAACATACTG | 54 |
|  | GABRG2Int5 | CCTACACATAAGAGCAGTCTC | GTATGGTCAAAGAGACATCATC | 58 |
|  | D5S2576 | TCGAGAGCCGTAATAACGC | GGGAAGGACTGCATAAAGC | 58 |
|  | D17S2131 | GGACATGCCACACACTG | CATGAGGGCAGATTTTCC | 58 |
|  | D5S422 | TTGATCGGGCTGGAGAAC | AACAGAGCAAGGTCCTGT | 58 |
| GEFS+4 | D2S1360 | GGCATATACAAAACAGAAACAG | CAGATTGTGGGACTTCTCAG | 58 |
|  | D2S305 | CTCCAGAGAAACAGAACCAAG | ACTCACCAGCCTCCATAATTG | 60 |
|  | D2S2342 | GAAGGCCAATGAGGAGAAAG | CAACGGCTCAACTGGGAAAG | 58 |

**Additional file 1:** *Table S2* *Primers Sequences of Microsatellite Markers for Haplotype Analysis on 22q13.31*

| Microsatellite | Band | Position (bp) | Genomic size (bp) | Primers Sequences | |
| --- | --- | --- | --- | --- | --- |
|  |  |  |  | Forward | Reverse |
| D22S1160 | 22q13.31 | 46429162–46429390 | 186-216 | CTTTGGGGAAGCAGTGAGTC | CCATGTTTGCAAAGGGAATC |
| D22S294 | 22q13.31 | 46951425-46951573 | 124 | GCATCCCAATTTAGGCCTTC | GCCTTATGGTGGCTTGTC |
| 15CAchr22a | 22q13.31 | 47157584-47157613 | 30 | GCAAACCAGACACGGTTT | AACTGCAGGTGCACACGTTA |
| 18GTchr22 | 22q13.31 | 47243816-47243852 | 36 | GACCAGTTCTGATGGCA | CAGTCTTCCCTCCGGGTATT |
| 17ACchr22 | 22q13.31 | 47479245-47479278 | 34 | TCAATTGCAAGACTCAGCAAA | CTTTGGGTTCATGTTTTGACTC |
| 15CAchr22b | 22q13.31 | 47771399-47772028 | 30 | AAATGGACTCCCCAAACACA | GCCAAGACCAGACAGTAGGC |
| 22ACchr22 | 22q13.31 | 48051336-48051380 | 44 | GGCAGATCTGGACTTGGTGT | GTCACTGTCCCTCTCTCTCCT |
| 21GTchr22 | 22q13.31 | 48252749-48252790 | 42 | ATGAGAAGGGGCACAAACTG | GGGGAAACAAAGCAGAAAAA |

***Additional file 1:*** *Table S3 Primers Sequences for Sanger Sequencing*

| Gene/Variant | Chromosome | Physical position (Mb) | Primer Sequences | |
| --- | --- | --- | --- | --- |
|  |  |  | Forward | Reverse |
| *KCNJ4*a | 22q13.1 | 38824274-38823659 | AGTCTTGGGGCTGAGTCTGA | GACCACCACAGCGATGACT |
| *KCNJ4*b | 22q13.1 | 38823905-38823342 | GGTGTATCGCCTTCTTCCAC | CAGGATGACCACGATCTCAA |
| *KCNJ4*c | 22q13.1 | 38823447-38822703 | CTATGACATCGGCCTGGAC | TTGGCTCTGTCCTGAGTGTG |
| *CACNA1I* T1478M | 22q13.31 | 39966758-40085742 | CTGCTCATCCACTCCATGTG | TTCCCTTCCAGACTCTGAGC |
| *TNRC6B* S1720L | 22q13.31 | 40440821-40731811 | TGGGACCTGCTCTGAATCTT | AAGGGAGGCAACTATGTGCT |
| *LDLRAP1* S202P | 1p36.11 | 25870071-25895377 | GTGATTGCTGGGGACAGAGT | CTGCCCACAGAGTGAGAACC |

**Additional file 1:** *Table S4* *Primers Sequences for HRM assay*

| Gene/Variant | Chromosome | Physical position (Mb) | Primer Sequences | |
| --- | --- | --- | --- | --- |
|  |  |  | Forward | Reverse |
| *CACNA1I* T1478M | 22q13.31 | 40.06 | CACCTTCATCATCTGCCTCA | CCCTCACCTTGTAACCTT |
| *TNRC6B* S1720L | 22q13.31 | 40.44 | TGGAAGATCCCCATAGG | TAGTGCTGCTGCTGTTCCAA |

**Additional file 1:** *Table S5 Lod Scores of linked locus on chromosome 22q13.31*

| Marker | Position on 22q13.31 (bp) | Lod Score |
| --- | --- | --- |
| rs3203726 | 37710013 | -11.4773 |
| rs138383 | 38799861 | 2.3719 |
| rs760645 | 39032270 | 2.404 |
| rs760482 | 39178701 | 2.42 |
| rs137636 | 39722477 | 2.453 |
| rs470084 | 40313356 | 2.4764 |
| rs139062 | 40881793 | 2.4921 |
| rs1884276 | 41143019 | 2.4991 |
| rs139451 | 41610024 | 2.5073 |
| rs2024566 | 41697338 | 2.5085 |
| rs80477 | 42001183 | 2.5111 |
| rs1052717 | 42281429 | 2.5129 |
| rs738377 | 43389570 | 2.5175 |
| rs139027 | 43649701 | 2.5182 |
| rs713912 | 44038518 | 2.519 |
| rs929090 | 44313849 | 2.5193 |
| rs12170546 | 44422333 | 2.5193 |
| D22S1140 | 44722825–44723195 | 2.5192 |
| rs763041 | 44763752 | 2.5191 |
| rs136904 | 45057826 | 2.5189 |
| rs1540327 | 45103486 | 2.5188 |
| D22S1168 | 45149978–45150286 | 2.5186 |
| D22S274 | 45269116–45269419 | 2.5185 |
| D22S928 | 45475302–45475721 | 2.5184 |
| rs132817 | 45532666 | 2.5182 |
| rs6006960 | 45648960 | 2.518 |
| D22S1141 | 45718826–45719171 | 2.5178 |
| D22S444 | 45961397–45961829 | 2.5176 |
| rs3213560 | 46022339 | 2.5174 |
| D22S532 | 46122948–46123462 | 2.5173 |
| rs714022 | 46175530 | 2.5172 |
| rs737822 | 46242791 | 2.517 |
| D22S1160 | 46429162–46429390 | 2.5165 |
| D22S294 | 46951425-46951573 | 2.5157 |
| rs5769127 | 47135272 | 2.5154 |
| 15CAchr22a | 47157584-47157613 | 2.5152 |
| 18GTchr22 | 47243816-47243852 | 2.515 |
| 17AC chr22 | 47479245-47479278 | 2.5148 |
| rs2017931 | 47566251 | 2.5146 |
| 15CAchr22b | 47771399-47772028 | 2.5144 |
| rs738486 | 47938564 | 1.9687 |
| 22ACchr22 | 48051036-48051680 | 1.9467 |
| 21GTchr22 | 48252449-48253090 | 1.9259 |
| rs728592 | 48271771 | -2.312 |

**Additional file 1:** *Table S6* *Loci on Chromosome 22q13.31 with Positive LOD Score*

| Physical Position (bp) | Size (bp) | Number of consecutive markers | Markers | Lod Score |
| --- | --- | --- | --- | --- |
| 38822333-38851205 | 28.87 | - | *KCNJ4* | - |
| 39032270 | 370 | 1 | rs760645 | 2.40 |
| 40881793 | 820 | 1 | rs139062 | 2.49 |
| 41610024 | 550 | 1 | rs139451 | 2.50 |
| 42001183 | 580 | 1 | rs80477 | 2.51 |
| 43389570-43649701 | 1750 | 2 | rs738377  rs139027 | 2.51  2.51 |
| 45532666 | 170 | 1 | rs132817 | 2.51 |

***Additional file 1:*** *Table S7 17ACchr22 Microsatellite Genotype and Allele Distributions in the Tunisian Control Population*

|  | n (%) |
| --- | --- |
| **Genotypes** | **n = 70** |
| (AC)13-(AC)19 | 1 (1.42) |
| (AC)14-(AC)17 | 1 (1.42) |
| (AC)14-(AC)18 | 1 (1.42) |
| (AC)15-(AC)15 | 1 (1.42) |
| (AC)15-(AC)16 | 1 (1.42) |
| (AC)15-(AC)17 | 2 (2.85) |
| (AC)15-(AC)19 | 2 (2.85) |
| (AC)15-(AC)20 | 4 (5.71) |
| (AC)15-(AC)21 | 1 (1.42) |
| (AC)16-(AC)17 | 1 (1.42) |
| (AC)16-(AC)18 | 2 (2.85) |
| (AC)16-(AC)19 | 4 (5.71) |
| (AC)16-(AC)20 | 1 (1.42) |
| (AC)16-(AC)21 | 1 (1.42) |
| (AC)17-(AC)17 | 5 (7.14) |
| (AC)17-(AC)19 | 5 (7.14) |
| (AC)17-(AC)20 | 4 (5.71) |
| (AC)18-(AC)18 | 2 (2.85) |
| (AC)18-(AC)19 | 4 (5.71) |
| (AC)18-(AC)20 | 2(2.85) |
| (AC)18-(AC)21 | 1 (1.42) |
| (AC)18-(AC)22 | 1 (1.42) |
| (AC)19-(AC)19 | 5 (7.14) |
| (AC)19-(AC)20 | 8 (11.42) |
| (AC)19-(AC)21 | 1 (1.42) |
| (AC)20-(AC)20 | 5 (7.14) |
| (AC)20-(AC)21 | 2 (2.85) |
| (AC)21-(AC)22 | 2 (2.85) |
| **Allele** | **n = 140** |
| AC13 | 1 (0.71) |
| AC14 | 2 (1.42) |
| AC15 | 12 (8.57) |
| AC16 | 10 (7.14) |
| AC17 | 25 (17.90) |
| AC18 | 15 (10.71) |
| AC19 | 35 (25) |
| AC20 | 31 (22.10) |
| AC21 | 8 (5.71) |
| AC22 | 1 (0.71) |
