# Supplementary figures and images for: A new locus on chromosome 22q13.31 linked to recessive genetic epilepsy with febrile seizures plus (GEFS+) in a Tunisian consanguineous family
Source: BMC Genet. 2013 Sep 25;14:93. doi: 10.1186/1471-2156-14-93 (PMC3851042; doi:10.1186/1471-2156-14-93)

## Slide 1
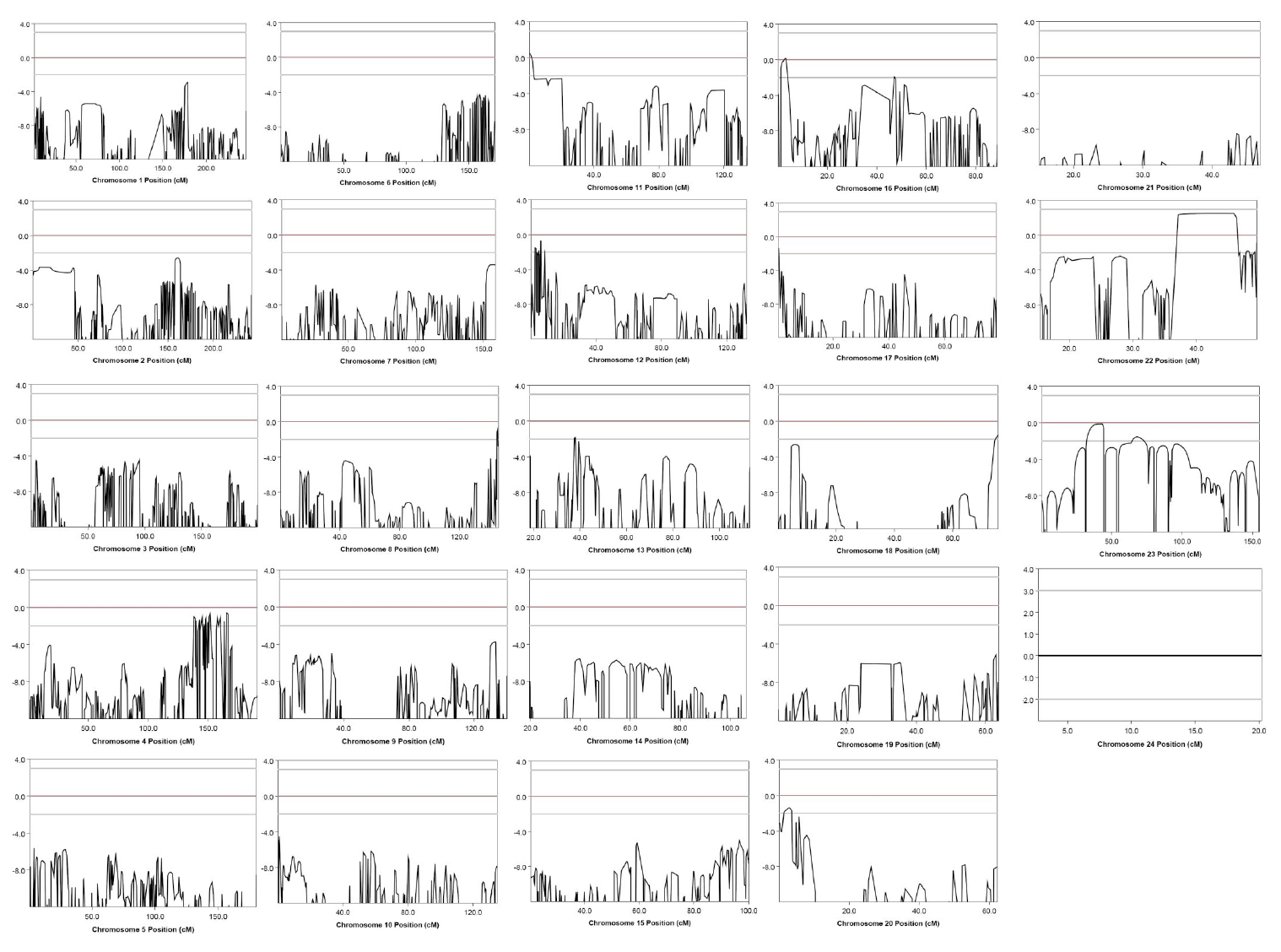

Supplement: Additional file 2: Figure FS1 — LOD scores were calculated using Merlin program. A new region was linked on the chromosome 22q with a maximum of LOD score of 2.51. [file 1471-2156-14-93-S2.ppt]
